# The association between neurodevelopmental and behavioral problems and tobacco smoke exposure among 3–17 years old children

**DOI:** 10.3389/fpubh.2022.881299

**Published:** 2022-08-10

**Authors:** Yu Gao, Tong Wang, Zhizhen Duan, Yuepu Pu, Juan Zhang

**Affiliations:** Key Laboratory of Environmental Medicine Engineering, Ministry of Education of China, School of Public Health, Southeast University, Nanjing, China

**Keywords:** tobacco smoke exposure, neurodevelopmental and behavioral problems, stratified analysis, children, age, gender

## Abstract

Children being exposed to tobacco smoke can lead to poor developmental and behavioral problems. We aimed to explore the correlation between neurodevelopmental and behavioral problems (NBPs) and tobacco smoke exposure (TSE) among children aged 3–17 years. In this study, data were obtained from the 2018–2019 U.S. National Survey of Children's Health (NSCH). Children in the range of 3–17 years old were taken as the research subjects, and their parents were surveyed through questionnaires. TSE status was defined as one of three groups: no tobacco smoke exposure (no TSE), someone smoking but not inside the house (no home TSE), and someone smoking inside the house (home TSE). NBPs mainly included behavioral or conduct problems, intellectual disability, learning disability, speech or other language disorders, and developmental delay. We used the sampling weights provided by the NSCH to weight the data in order to obtain an unbiased population estimate. One-way ANOVA and Chi-square tests were performed to examine the difference of each variable. Logistic regression analysis and stratified analysis were carried out to investigate the association between NBPs and TSE. A total of 48,783 children were included in this study, with an average age of 10.1 years. In total 17.9% of all the participants were preschool children, 35.1% were school-age children, and 47.0% were school-age adolescents. More than 85.0% of children lived with no TSE. Over 90.0% of children were healthy in each NBP. Children living with home TSE and no home TSE showed significant adjusted odds ratios (aORs) compared with no TSE in four NBPs besides intellectual disability. The stratified analysis found aORs were higher for NBPs in preschool children compared to the school-age children and school-age adolescents. Male children living with home TSE showed higher aORs in moderate/severe NBP conditions. Our study indicated it is necessary to protect the health of young children from TSE by intervention measures.

## Introduction

Tobacco smoke exposure (TSE) is well-known to be an important public health issue. Tobacco use can not only contribute to 6 million deaths every year worldwide, but the health damages are not limited to smokers themselves, but also to non-smokers in the vicinity (1). There are no safe exposure levels for the TSE (2). TSE in children mainly includes secondhand smoke (SHS) and thirdhand smoke (THS) exposure. SHS is derived from the smoke produced when others burn tobacco, including the main-stream smoke emitted by the smoker and side-stream smoke released during cigarette burning (3). THS refers to tobacco smoke contaminants remaining on furniture, walls, hair and skin surfaces, and dust after smoking (4). THS originates from SHS and persists for a long time, it can be re-released into the environment for days or even months. Previous studies have suggested that THS may be more harmful than SHS exposure (5). Children are often exposed to second- and third-hand smoke in an indoor environment at the same time.

According to the Diagnostic and Statistical Manual of Mental Disorders 5th edition (DSM-5), neurodevelopmental disorders are a group of conditions with onset in the developmental period. The disorders typically manifest before the child enters grade school, and are characterized by developmental deficits (6). Neurodevelopmental and behavioral problems (NBPs) principally include developmental delay, motor disorder, intellectual disability, language disorder, autism spectrum disorder, specific learning disability, and attention-deficit/hyperactivity disorder (7, 8). Neurodevelopmental disorders frequently co-occur, for example, many children with attention-deficit/hyperactivity disorder also have a specific learning disorder.

Children were reported to be more sensitive and vulnerable to TSE than adults because of their special metabolic characteristics (4, 9). A Chinese birth cohort study found infants exposed to tobacco smoke were associated with upper respiratory tract infections (10). Liu et al. provided the first characterization of the relationship between urine 4-(methylnitrosamino)-1-(3-pyridyl)-1-butanol and blood pressure z-scores in children with SHS (11). Adverse behavioral and cognitive outcomes were also associated with smoke exposure in children (12–14). A dose–response relationship was found between levels of SHS and behavioral problems (15). A Chinese prospective cohort indicated that exposure to the polycyclic aromatic hydrocarbons (PAHs) produced by tobacco smoke during pregnancy can lead to severe health damage (16).

The chemical composition of tobacco smoke is very complicated, thousands of compounds have been identified, among them tar, nicotine, and PAHs. The potential mechanisms of tobacco smoke on cognition were investigated in a previous study (17). A study found an increase of reactive oxygen species and nitric oxide synthase in the brain of rats after exposure to cigarettes, which resulted in DNA breakage and ultrastructural changes of the cerebral cortex (18). Tobacco smoke could activate redox-sensitive transcription factors, which in turn induced the release of proinflammatory mediators from cells (19). Tobacco smoke could not only change the morphology and function of endothelial cells, but also enhanced the activity of platelets and accelerated the formation of atherosclerosis (20).

Most of the previous studies have only explored the relationship between TSE and NBPs in some age groups but did not cover all the age groups of children. A study based on a larger sample size and covering preschool children, school-age children, and adolescents needs to be performed to reveal the risks of NBPs caused by TSE in age and gender. Thus, this study aimed to explore the correlation between TSE and NBPs in children aged 3–17 years, and especially to investigate the impact of different age and gender groups. Our results provided further evidence for protecting the neurodevelopment in children.

## Methods

### Study population

The data for this study were obtained from the 2018–2019 U.S. National Survey of Children's Health (NSCH). The NSCH has mainly focused on the mental and physical health of 0–17 year old children in the United States. Addresses were randomly selected by the NSCH from families across the United States and adults who knew the child's health and health care (usually the parent) were invited to complete a brief screening questionnaire *via* the internet or on paper to identify if there are children aged 0–17 years in the respondent's home. In the case of multiple children aged 0–17 years in a home, one of the children was randomly selected as the investigation subject for this survey to enter the age-appropriate topical questionnaire. The 2018–2019 NSCH collected information on a total of 59,963 children completed. In total, 30,530 surveys were completed in 2018 and 29,433 in 2019. The overall weighted response rate was 43.1% for 2018 and 42.4% for 2019. The 2018 and 2019 combined data set contains approximately 1,176 surveys per state (state range: 1,021 to 1,420). We finally included children to children aged 3–17 years in our analysis (*N* = 48783), excluding children aged 0–2 years (*n* = 7,427), missing data on tobacco smoke exposure (*n* = 801) and neurodevelopmental conditions (*n* = 26), child and family characteristics (*n* = 2,781).

### Study measures

The status of TSE in children was derived from parents/caregivers responses to the question “Does anyone living in your household use cigarettes, cigars, or pipe tobacco?” and “Does anyone smoke inside your home?” The two questions were combined into one variable for analysis and finally, three types of TSE were confirmed: no TSE, household smoker with no home TSE (no home TSE), and household smoker with home TSE (home TSE).

Neurodevelopmental and behavioral problems were composed of five items. Parents/caregivers reported whether any professional staff ever affirmed the following conditions of their child (yes/no): (1) intellectual disability, (2) behavioral or conduct problems, (3) speech or other language disorder, (4) developmental delay, and (5) learning disability. If yes to any of these items, then they were required to respond to whether the child currently had the condition. If so, parents/caregivers selected the severity of the problems (mild, moderate, or severe). We considered the following sociodemographic characteristics as covariates: child age, sex, race, health status, premature birth, and low birth weight.

### Statistical analysis

SPSS statistical software (version 24.0) was used for the data collation and analysis. We used the sampling weights provided by NSCH to weight the data in order to obtain an unbiased population estimate. We performed a descriptive statistical analysis of all the variables and listed frequency and weighted percent. One-way ANOVA and Chi-square tests were conducted to examine the difference of each variable. Then, univariate and multivariable logistic regression analyses were used to investigate the correlation between NBPs and TSE status. Furthermore, stratified analysis by age (preschool children: 3–5 years old; school-age children: 6–11 years old; school-age adolescents: 12–17 years old) and gender (male and female) was performed. All the aforementioned analyses were two-sided tests, and *P* < 0.05 was statistically significant.

## Results

### Study characteristics by TSE status

The NSCH included weight data for 48,783 children 3–17 years of age for the year 2018–2019 (Table 1). The total mean age of children was 10.1 (SD = 4.3) years, of which 17.9% were preschool children, 35.1% were school-age children, and 47.0% were school-age adolescents. In this stufy 51.3% were boys. Over half (51.3%) were non-Hispanic white, and the ethnicity with the lowest representation in the study was Hispanic (10.6%). Approximately 90% of children had excellent or very good health, while 11.5% of children were premature births and 9.1% had low birth weight. More than 85% of children lived with no TSE. The younger the age group was, the lower the percent of home TSE was. The highest percentage of children with no TSE were Hispanic (88.1%), and the group with the highest home TSE was Non-Hispanic black (3.8%). There were also significant differences in child health status, premature birth, and low birth weight by different TSE status.

**Table 1:** Characteristics of children aged 3–17 years by TSE status, 2018-2019 NSCH.

**Characteristic**	**Overall** **(*N* = 48,783),** ***n* (%)^a^**	**TSE status**			***P*-Value**
		**No TSE** **(*N* = 41,588),** ***n* (%) ^b^**	**Household smoker—no home TSE** **(*N* = 6,278), *n* (%) ^b^**	**Household smoker—home TSE** **(*N* = 917), *n* (%) ^b^**	
Child age, Mean (SD)	10.1 (4.3)	10.0 (4.3)	10.2 (4.3)	11.2 (4.1)	<0.001
**Age group**					<0.001
3–5years	8751 (17.9)	7602 (86.9)	1062 (12.1)	87 (1.0)	
6–11years	171066 (35.1)	14567 (85.1)	2252 (13.2)	287 (1.7)	
12–17years	22926 (47.0)	19419 (84.7)	2964 (12.9)	543 (2.4)	
**Child gender**					<0.001
Male	25427 (51.3)	21684 (85.4)	3259 (12.8)	484 (1.8)	
Female	23356 (48.7)	19904 (85.3)	3019 (12.6)	433 (2.1)	
**Child race**					<0.001
Non-Hispanic white	34311 (51.3)	29069 (83.7)	4606 (14.2)	636 (2.1)	
Non-Hispanic black	2971 (13.0)	2551 (86.5)	304 (9.7)	116 (3.8)	
Hispanic	5639 (25.1)	4938 (88.1)	651 (11.4)	50 (0.5)	
Non-Hispanic other/multi-racial	5862 (10.6)	5030 (85.3)	717 (12.4)	115 (2.3)	
**Child health status**					<0.001
Excellent or very good	44678 (89.8)	38396 (86.1)	5532 (12.1)	750 (1.8)	
Good	3473 (8.6)	2698 (78.2)	632 (18.3)	143 (3.5)	
Fair or poor	632 (1.6)	494 (78.8)	114 (17.4)	24 (3.8)	
**Child premature birth**					<0.001
Yes	5424 (11.5)	4461 (82.6)	824 (14.9)	139 (2.5)	
No	43359 (88.5)	37127 (85.7)	5454 (12.4)	778 (1.9)	
**Low birth weight**					<0.001
Yes	4130 (9.1)	3372 (82.1)	631 (14.4)	127 (3.4)	
No	44653 (90.9)	38216 (85.6)	5647 (12.6)	790 (1.8)	

NSCH, National Survey on Children's Health; TSE, tobacco smoke exposure.

^a^n (%) refers to raw sample size and weighted column percentage; ^b^n (%) refers to raw sample size and weighted row percentage.

The results of the NBPs are shown in Table 2. Over 90.0% of children were healthy in each NBP. The highest percentage of moderate/severe behavioral or conduct problems (3.9%) were found among five moderate/severe problems. NBPs seemed to get worse with home TSE. Significant differences were shown in all five problems by TSE status.

**Table 2:** Prevalence of NBPs by different status of TSE among children aged 3–17 years, 2018-2019 NSCH.

**Characteristic**	**Overall** ***N* (%)^a^**	**TSE status**			***P*-Value**
		**No TSE** ***n* (%)^b^**	**Household smoker—no home TSE** ***n* (%) ^b^**	**Household smoker—home TSE** ***n* (%) ^b^**	
**Behavioral or conduct problems (*****N*** **= 48,570)**					<0.001
No condition	45144 (93.2)	38875 (86.3)	5530 (12.0)	739 (1.7)	
Mild	1531 (2.9)	1203 (76.1)	264 (19.6)	64 (4.3)	
Moderate/Severe	1895 (3.9)	1338 (69.5)	454 (24.2)	103 (6.3)	
**Developmental delay (*****N*** **= 48,548)**					<0.001
No condition	45998 (94.8)	39391 (85.8)	5803 (12.4)	804 (1.8)	
Mild	1171 (2.3)	930 (74.1)	195 (21.4)	46 (4.5)	
Moderate/Severe	1379 (2.9)	1086 (79.7)	236 (16.4)	57 (3.9)	
**Intellectual disability (*****N*** **= 48,656)**					<0.001
No condition	48166 (99.0)	41089 (85.3)	6178 (12.7)	899 (2.0)	
Mild	163 (0.3)	124 (73.4)	35 (23.8)	4 (2.8)	
Moderate/Severe	327 (0.7)	272 (86.7)	44 (10.5)	11 (2.8)	
**Speech or other language disorder (*****N*** **= 48,613)**					<0.001
No condition	46059 (94.4)	39378 (85.7)	5850 (12.4)	831 (1.9)	
Mild	1528 (3.3)	1237 (78.5)	239 (18.6)	52 (2.8)	
Moderate/Severe	1026 (2.3)	831 (80.6)	164 (16.1)	31 (3.3)	
**Learning disability (*****N*** **= 48,612)**					<0.001
No condition	45191 (93.5)	38735 (85.8)	5669 (12.4)	787 (1.8)	
Mild	1694 (3.1)	1367 (77.8)	272 (18.6)	55 (3.6)	
Moderate/Severe	1727 (3.4)	1350 (78.9)	311 (18.1)	66 (3.0)	

NSCH, National Survey on Children's Health; TSE, tobacco smoke exposure; NBPs, neurodevelopmental and behavioral problems.

^a^n (%) refers to raw sample size and weighted column percentage; ^b^n (%) refers to raw sample size and weighted row percentage. Missing values excluded.

### Logistic regression analysis of TSE status and NBPs

**Figure 1:**
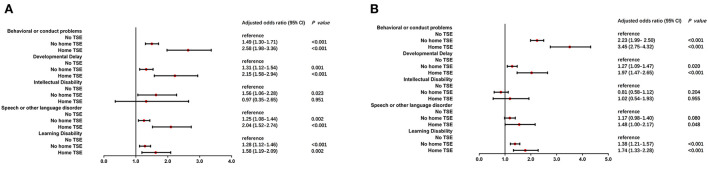
Multiple logistic regression analysis of TSE and NBPs. After adjusted for child age, gender, race, health status, premature birth and low birth weight, children who lived with/without home TSE (home TSE/no home TSE) compared to children who lived with no TSE (no TSE, reference) in mild **(A)** and moderate/severe **(B)** condition of NBPs.

Univariate logistic regression analysis indicated that children living with no home TSE and with home TSE had higher odds ratios (ORs) to result in NBPs compared to children with no TSE (Supplementary Table 1). Intellectual disability was not found to show significant results among the top five problems caused by TSE status. Increased odds were found in children living with home TSE on speech or other language disorders, behavioral or conduct problems, developmental delay, and learning disability (*P* < 0.05). The results of multiple logistic regression analysis provided more evidence (Figure 1). After adjusting for child age, gender, race, health status, premature birth, and low birth weight, similar results were found compared to the unadjusted model. Four NBPs showed significant adjusted odds ratios (aORs) besides intellectual disability. Children who lived with home TSE were respectively, 2.58 (95% CI = 1.98–3.36) and 3.45 (95% CI = 2.75–4.32) times more likely than children with no TSE to have mild and moderate/severe behavioral or conduct problems. Meanwhile, Children living with no home TSE were respectively, 1.49 (95% CI = 1.30–1.71) and 2.23 (95% CI = 1.99–2.50) times more likely to children with no TSE to have mild and moderate/severe behavioral or conduct problems.

### Stratified analysis by age and gender

Stratified analysis for age and gender was performed to investigate further correlation between TSE and NBPs. Children were divided into three subgroups by age, preschool children, school-age children, and school-age adolescents. Two subgroups male and female were separated by gender.

**Figure 2:**
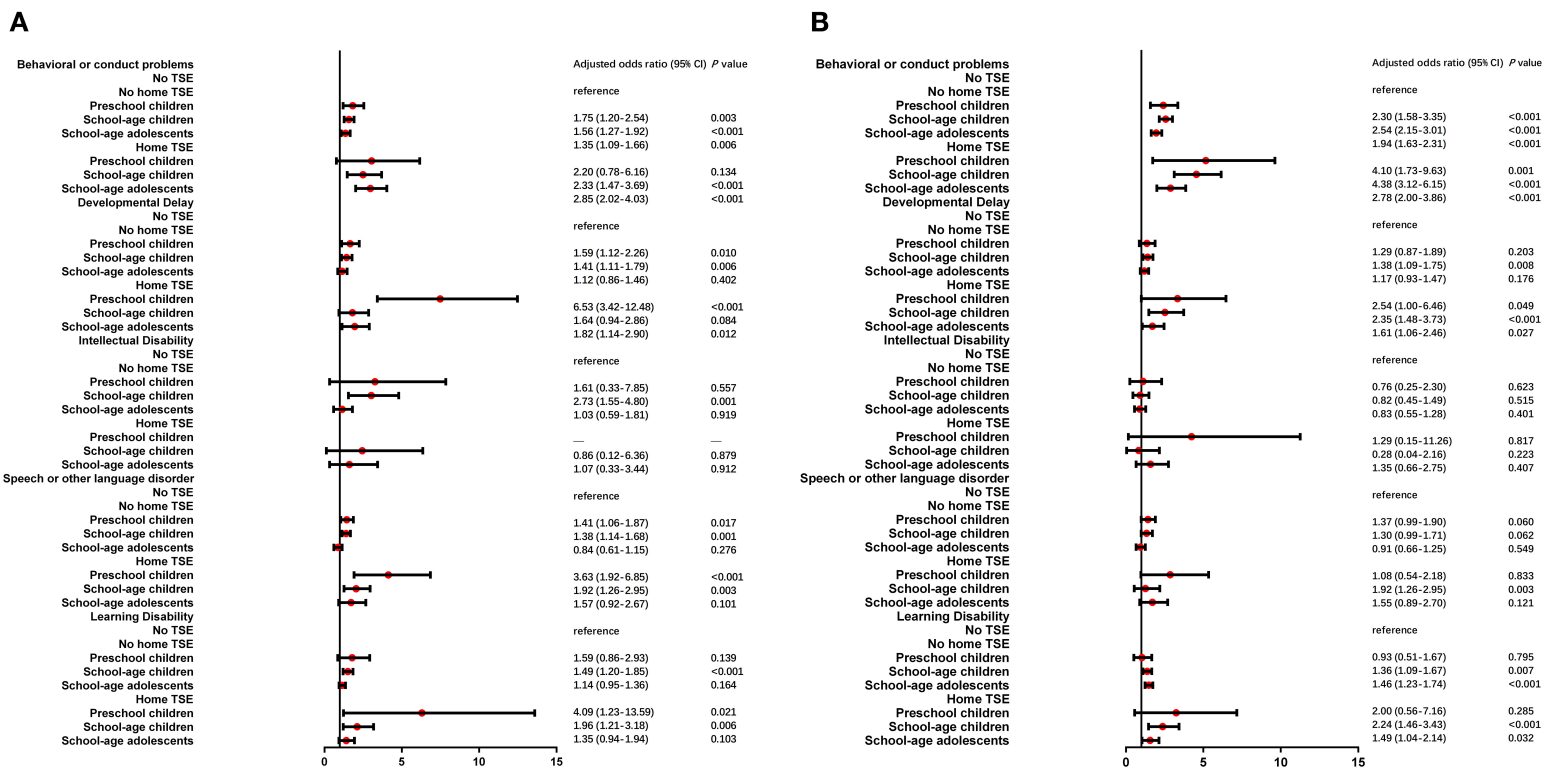
Multiple logistic regression analysis of TSE and NBPs by age stratified analysis. After adjusted for child age, gender, race, health status, premature birth and low birth weight, three groups of children (preschool children, school-age children and school-age adolescents) who lived with/without home TSE (home TSE/no home TSE) compared to children who lived with no TSE (no TSE, reference) in mild **(A)** and moderate/severe **(B)** condition of NBPs.

An unadjusted model was carried out to investigate the association between TSE and neurodevelopmental and behavioral problems in different age subgroups (Supplementary Table 2). Children living with home TSE showed the highest odds ratios and were more likely than children with no TSE to suffer mild neurodevelopmental and behavioral problems in the preschool children group (Behavioral or conduct problems: OR = 2.53, developmental delay: OR = 7.10, learning disability: OR = 5.07 and speech or other language disorder: OR = 3.95). Intellectual disability was still not significant in all three age subgroups. Then multivariable logistic regression analysis was performed in Figure 2. Developmental delay, learning disability, and speech or other language disorders found the highest odds ratios for mild conditions in the preschool children group. aORs were closer between preschool children and school-aged children in moderate/severe conditions, and the lowest aOR was in school-aged adolescents after adjustment.

**Figure 3:**
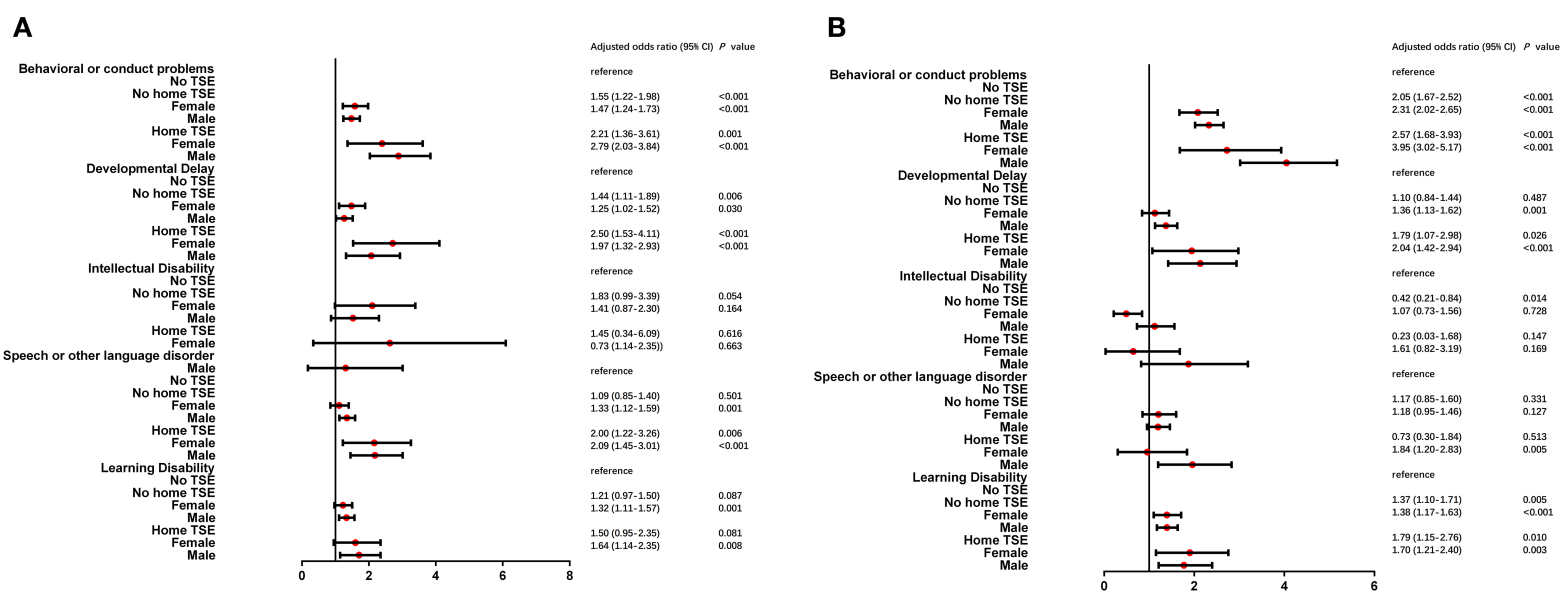
Multiple logistic regression analysis of TSE and NBPs by gender stratified analysis. After adjusted for child age, gender, race, health status, premature birth and low birth weight, two groups of children (male and female) who lived with/without home TSE (home TSE/no home TSE) compared to children who lived with no TSE (no TSE, reference) in mild **(A)** and moderate/severe **(B)** condition of NBPs.

Univariate logistic regression stratified by gender is shown in Supplementary Table 3. ORs of children with home TSE were higher than those of children with no TSE among five neurodevelopmental and behavioral problems. In moderate/severe conditions, ORs were higher in boys with home TSE. After adjusting for child age, race, health status, premature birth, and low birth weight, the results were found in Figure 3. Children with home TSE were higher aORs in moderate/severe conditions in boys, the most notable of which were behavioral or conduct problems (aOR: 3.95 vs. 2.57) and developmental delay (aOR: 2.04 vs. 1.79).

## Discussion

In this study, we investigated the status of TSE and NBPs in children aged 3–17 years based on the data from the 2018–2019 NSCH. The results showed children with home TSE increased the risks of NBPs. Preschool children were more sensitive to NBPs caused by tobacco exposure. Children with home TSE showed higher aORs of behavioral or conduct problems and developmental delay in boys.

The result of this study showed that the percentage of TSE at home in children was 14.7%, of which 12.7% lived with no home TSE and 2.0% lived with home TSE. Tsai et al. found SHS exposure declined from approximately 90.0 to 40.0% among children and adolescents from 1988 to 2014 (21). Compared to our results, the ratio of smoke exposure in children has been further reduced. This may be related to smoke-free home policies issued by the US government as well as the implementation among US adults (22, 23). But we still found that children aged 6–17 years showed higher rates of TSE at home than children aged 3–5 years, which was consistent with previous findings (22, 24). Therefore, the implementation of household smoke-free policies should be further enhanced, and this was meaningful for the neurodevelopmental health of children in public health.

The overall weighted prevalence of NBPs in children was: behavioral or conduct problems (6.8%), developmental delay (5.2%), intellectual disability (1.0%), learning disability (6.5%), and speech or other language disorder (5.6%). The percentages of all five NBPs showed a decrease compared with the results of the study carried out in 2016–2017 (25). The prevalence of the five NBPs showed significant differences among the three statuses of TSE (*P* < 0.001). This revealed TSE in children had an effect on the occurrence of NBPs. A previous study found a correlation between SHS exposure in the home and mental disorders (26). Max et al. revealed SHS exposure in children was strongly associated with attention deficit hyperactivity disorder (ADHD) independent of other risk factors (27). The level of serum cotinine was showed significantly associated with depressive and conduct disorder in 8–15 years old children (28).

The results of logistic the regression found the ORs of NBPs caused by the status of TSE in our study. Our results indicated that after adjusting for confounding factors, TSE at home still had a significant impact on NBPs in children besides intellectual disability. Children living with home TSE had higher aORs compared with the children living with no home TSE. It confirmed THS exposure which also resulted in the health damage. Exposure to THS can lead to the weight of neonatal mice being less than non-exposed mice and THS also can produce continuous changes in the hematopoietic system (29). Cancer risk assessment modeling studies indicated that nitrosamines in THS may increase the risk of cancer in non-smokers. And it is suggested that indoor dust may be the main way for children and non-smokers to be exposed to nitrosamines (30). The results may be due to the former only comprising a separate exposure to SHS, while the latter contains a mixed exposure to SHS and THS. The stratified analysis further revealed the effect of age and gender on NBPs in children. The results showed preschool children were at higher risk of behavioral and conduct problems. A previous study found that younger children (age 6–8 years) had higher ORs of neurodevelopmental conditions than older children (age 9–11 years) (25). The effects of TSE at home on NBPs in boys were more pronounced. The different risks caused by gender could be further investigated by more epidemiologic studies.

## Conclusion

Our study revealed children living with TSE at home were associated with four neurodevelopmental and behavioral problems, namely, behavioral or conduct problems, developmental delay, learning disability, and speech or other language disorder. The higher risks of NBPs caused by TSE were found in preschool and male children. There still needs to be more quantitative research to clarify the association between the tobacco exposure level and neurodevelopmental and behavioral problems in children. Therefore, it is very important to ensure no TSE at home and in public places for the growth and development of infants and children.

## Limitations

Although this study had a large sample size and representative sample, several limitations remain. First, this is a cross-sectional study and causality could not be determined. Second, the variables in the study were derived from parents/caregivers reports rather than objective markers that accurately respond to tobacco smoke exposure, and thus, there may be reporting bias. In addition, although some variables were adjusted in this study, it did not account for maternal smoking and passive smoking during pregnancy, and cumulative tobacco smoke exposure in children was not assessed. However, there are studies that using objective markers of TSH exposure to assess children's exposure after excluding active and passive smoking during pregnancy in mothers, indicating that the results obtained are largely valid in this study.

## Data availability statement

The original contributions presented in the study are included in the article/Supplementary material, further inquiries can be directed to the corresponding author.

## Ethics statement

This study used de-identified data and as such was not considered to be human subjects research.

## Author contributions

Conceptualization and writing–original draft preparation: YG. Formal analysis and investigation: TW and ZD. Validation and writing–review and editing: TW, YP, and JZ. All authors contributed to the article and approved the submitted version.

## Conflict of interest

The authors declare that the research was conducted in the absence of any commercial or financial relationships that could be construed as a potential conflict of interest.

## Publisher's note

All claims expressed in this article are solely those of the authors and do not necessarily represent those of their affiliated organizations, or those of the publisher, the editors and the reviewers. Any product that may be evaluated in this article, or claim that may be made by its manufacturer, is not guaranteed or endorsed by the publisher.
