# Supplementary material for: The association between neurodevelopmental and behavioral problems and tobacco smoke exposure among 3–17 years old children
Source: Front Public Health. 2022 Aug 10;10:881299. doi: 10.3389/fpubh.2022.881299 (PMC9399492; doi:10.3389/fpubh.2022.881299)
Supplement: Supplementary file 1 [file Table_1.DOCX]

Supplement Table 1. univariate logistic regression analysis of TSE and NBPs

| Variables | Univariate regression | | | | | |
| --- | --- | --- | --- | --- | --- | --- |
|  | Mild | | | Moderate/Severe | | |
|  | OR | 95%CI | *P* -Value | OR | 95%CI | *P* -Value |
| Behavioral or conduct problems |  |  |  |  |  |  |
| No TSE | Reference | | | | | |
| No home TSE | 1.543 | (1.346-1.768) | <0.001 | 2.385 | (2.137-2.663) | <0.001 |
| Home TSE | 2.799 | (2.154-3.636) | <0.001 | 4.050 | (3.272-5.012) | <0.001 |
| Developmental Delay |  |  |  |  |  |  |
| No TSE | Reference | | | | | |
| No home TSE | 1.423 | (1.217-1.665) | <0.001 | 1.475 | (1.278-1.703) | <0.001 |
| Home TSE | 2.423 | (1.788-3.285) | <0.001 | 2.571 | (1.953-3.387) | <0.001 |
| Intellectual Disability |  |  |  |  |  |  |
| No TSE | Reference | | | | | |
| No home TSE | 1.877 | (1.289-2.734) | 0.001 | 1.076 | (0.782-1.481) | 0.654 |
| Home TSE | 1.474 | (0.544-3.999) | 0.446 | 1.848 | (1.008-3.390) | 0.047 |
| Speech or other language disorder |  |  |  |  |  |  |
| No TSE | Reference | | | | | |
| No home TSE | 1.301 | (1.129-1.498) | <0.001 | 1.328 | (1.121-1.574) | 0.001 |
| Home TSE | 1.992 | (1.497-2.651) | <0.001 | 1.768 | (1.227-2.547) | 0.002 |
| Learning Disability |  |  |  |  |  |  |
| No TSE | Reference | | | | | |
| No home TSE | 1.360 | (1.190-1.553) | <0.001 | 1.574 | (1.387-1.786) | 0.001 |
| Home TSE | 1.980 | (1.499-2.617) | <0.001 | 2.406 | (1.861-3.111) | 0.002 |

Note: TSE = tobacco smoke exposure; OR = odds ratio; CI = confidence interval; NBPs = neurodevelopmental and behavioral problems

Supplement Table 2. Univariate logistic regression of TSE and NBPs by age stratified analysis

| Variables | Preschool children（3-5 years old） | | | | School-age children（6-11 years old） | | | | School-age adolescents（12-17 years old） | | | |
| --- | --- | --- | --- | --- | --- | --- | --- | --- | --- | --- | --- | --- |
|  | Mild | | Moderate/Severe | | Mild | | Moderate/Severe | | Mild | | Moderate/Severe | |
|  | OR (95%CI) | *P* -Value | OR (95% CI) | *P* -Value | OR (95% CI) | *P* -Value | OR (95% CI) | *P*-Value | OR (95% CI) | *P* -Value | OR (95% CI) | *P* -Value |
| Behavioral or conduct problems |  |  |  |  |  |  |  |  |  |  |  |  |
| No TSE | Reference | | | | | | | | | | | |
| No home TSE | 1.76 (1.22-2.54) | 0.003 | 2.36 (1.64-3.38) | <0.001 | 1.55 (1.27-1.91) | <0.001 | 2.53 (2.15-2.97) | <0.001 | 1.46 (1.19-1.80) | <0.001 | 2.23 (1.89-2.63) | <0.001 |
| Home TSE | 2.53 (0.91-7.00) | 0.074 | 4.30 (2.41-11.81) | <0.001 | 2.51 (1.59-3.96) | <0.001 | 5.08 (3.69-7.01) | <0.001 | 3.01 (2.15-4.22) | <0.001 | 3.14 (2.30-4.29) | <0.001 |
| Developmental Delay |  |  |  |  |  |  |  |  |  |  |  |  |
| No TSE | Reference | | | | | | | | | | | |
| No home TSE | 1.64 (1.16-2.33) | 0.005 | 1.40 (0.97-2.01) | 0.070 | 1.49 (1.17-1.89) | 0.001 | 1.50 (1.19-1.88) | <0.001 | 1.26 (0.97-1.63) | 0.083 | 1.48 (1.19-1.84) | <0.001 |
| Home TSE | 7.10 (3.78-13.35) | <0.001 | 3.36 (1.44-7.84) | 0.005 | 2.02 (1.17-3.50) | 0.012 | 3.12 (2.03-4.79) | <0.001 | 2.06 (1.30-3.26) | 0.002 | 2.17 (1.46-3.24) | <0.001 |
| Intellectual Disability |  |  |  |  |  |  |  |  |  |  |  |  |
| No TSE | Reference | | | | | | | | | | | |
| No home TSE | 1.80 (0.38-8.48) | 0.458 | 0.99 (0.35-2.83) | 0.989 | 3.00 (1.71-5.26) | <0.001 | 0.94 (0.52-1.68) | 0.833 | 1.28 (0.74-2.23) | 0.384 | 1.16 (0.77-1.75) | 0.481 |
| Home TSE | —— | —— | 3.03 (0.41-22.47) | 0.279 | 1.30 (0.18-9.49) | 0.796 | 0.56 (0.08-4.06) | 0.569 | 1.41 (0.44-4.48) | 0.560 | 2.13 (1.08-4.19) | 0.029 |
| Speech or other language disorder |  |  |  |  |  |  |  |  |  |  |  |  |
| No TSE | Reference | | | | | | | | | | | |
| No home TSE | 1.45 (1.09-1.92) | 0.010 | 1.45 (1.06-1.98) | 0.021 | 1.43 (1.18-1.74) | <0.001 | 1.40 (1.08-1.83) | 0.013 | 0.97 (0.71-1.32) | 0.829 | 1.20 (0.88-1.64) | 0.262 |
| Home TSE | 3.95 (2.12-7.38) | <0.001 | 2.92 (1.33-6.42) | <0.001 | 2.20 (1.45-3.34) | <0.001 | 1.51 (0.77-2.95) | 0.234 | 1.80 (1.06-3.04) | 0.029 | 2.13 (1.26-3.62) | 0.005 |
| Learning Disability |  |  |  |  |  |  |  |  |  |  |  |  |
| No TSE | Reference | | | | | | | | | | | |
| No home TSE | 1.70 (0.93-3.12) | 0.087 | 1.10 (0.64-1.90) | 0.730 | 1.56 (1.26-1.93) | <0.001 | 1.43 (1.16-1.76) | 0.001 | 1.21 (1.01-1.44) | 0.038 | 1.72 (1.46-2.04) | <0.001 |
| Home TSE | 5.07 (1.55-16.54) | 0.007 | 2.84 (0.88-9.16) | 0.080 | 2.30 (1.43-3.71) | 0.001 | 2.87 (1.91-4.31) | <0.001 | 1.48 (1.04-2.13) | 0.032 | 1.90 (1.34-2.69) | <0.001 |

Note: TSE = tobacco smoke exposure; OR= odds ratio; CI = confidence interval; NBPs = neurodevelopmental and behavioral problems

Supplement Table 3. Univariate logistic regression of TSE and NBPs by gender stratified analysis

| Variables | Female | | | | | | Male | | | | | | |
| --- | --- | --- | --- | --- | --- | --- | --- | --- | --- | --- | --- | --- | --- |
|  | Mild | | | Moderate/severe | | | | Mild | | | Moderate/severe | | |
|  | OR | 95%CI | *P*-Value | OR | 95%CI | *P*-Value | | OR | 95%CI | *P*-Value | OR | 95%CI | *P*-Value |
| Behavioral or conduct problems |  |  |  |  |  |  | |  |  |  |  |  |  |
| No TSE | Reference | | | | | | | | | | | | |
| No home TSE | 1.65 | (1.30-2.10) | <0.001 | 2.26 | (1.85-2.76) | <0.001 | | 1.52 | (1.29-1.79) | <0.001 | 2.48 | (2.17-2.83) | <0.001 |
| Home TSE | 2.56 | (1.58-4.15) | <0.001 | 3.24 | (2.15-4.88) | <0.001 | | 3.01 | (2.20-4.13) | <0.001 | 4.58 | (3.55-5.92) | <0.001 |
| Developmental Delay |  |  |  |  |  |  | |  |  |  |  |  |  |
| No TSE | Reference | | | | | | | | | | | | |
| No home TSE | 1.61 | (1.24-2.08) | <0.001 | 1.34 | (1.03-1.73) | 0.029 | | 1.34 | (1.10-1.63) | 0.004 | 1.55 | (1.31-1.84) | <0.001 |
| Home TSE | 2.92 | (1.80-4.74) | <0.001 | 2.50 | (1.54-4.05) | <0.001 | | 2.19 | (1.48-3.24) | <0.001 | 2.61 | (1.87-3.66) | <0.001 |
| Intellectual Disability |  |  |  |  |  |  | |  |  |  |  |  |  |
| No TSE | Reference | | | | | | | | | | | | |
| No home TSE | 2.25 | (1.23-4.14) | 0.009 | 0.58 | (0.29-1.14) | 0.113 | | 1.70 | (1.05-2.74) | 0.031 | 1.39 | (0.96-2.00) | 0.079 |
| Home TSE | 2.24 | (0.54-9.29) | 0.266 | 0.45 | (0.06-3.20) | 0.422 | | 1.10 | (0.27-4.47) | 0.898 | 2.69 | (1.41-5.13) | 0.003 |
| Speech or other language disorder |  |  |  |  |  |  | |  |  |  |  |  |  |
| No TSE | Reference | | | | | | | | | | | | |
| No home TSE | 1.17 | (0.91-1.50) | 0.220 | 1.37 | (1.01-1.87) | 0.042 | | 1.38 | (1.16-1.64) | <0.001 | 1.32 | (1.07-1.62) | 0.008 |
| Home TSE | 1.98 | (1.22-3.21) | 0.005 | 0.97 | (0.40-2.37) | 0.948 | | 2.00 | (1.40-2.86) | <0.001 | 2.11 | (1.41-3.16) | <0.001 |
| Learning Disability |  |  |  |  |  |  | |  |  |  |  |  |  |
| No TSE | Reference | | | | | | | | | | | | |
| No home TSE | 1.29 | (1.04-1.59) | 0.020 | 1.59 | (1.29-1.97) | <0.001 | | 1.41 | (1.19-1.68) | <0.001 | 1.58 | (1.35-1.84) | <0.001 |
| Home TSE | 1.89 | (1.21-2.96) | 0.005 | 2.57 | (1.69-3.92) | <0.001 | | 2.04 | (1.43-2.92) | <0.001 | 2.33 | (1.68-3.22) | <0.001 |

Note: TSE = tobacco smoke exposure; OR= odds ratio; CI = confidence interval; NBPs = neurodevelopmental and behavioral problems
